# Biotechnology of Edible Fungi

**DOI:** 10.3390/jof9101025

**Published:** 2023-10-19

**Authors:** Gen Zou, Jing Zhu, Mingwen Zhao

**Affiliations:** 1 Institute of Edible Fungi, Shanghai Academy of Agricultural Sciences, Shanghai 201403, China; zougen@sibs.ac.cn; 2 College of Life Sciences, Nanjing Agricultural University, Nanjing 210095, China; jingzhu@njau.edu.cn

Edible fungi are generally defined as macrofungi with large fruiting bodies that may be consumed by humans and are commonly referred to as mushrooms. As a valuable source of proteins, fibers, minerals, vitamins, and other bioactive compounds, mushrooms have been consumed by humans for cultural, medicinal, recreational, and religious purposes, extending beyond their use as food for centuries. A typical fungal cell has precisely two genetically distinct but allelically compatible nuclei. When cell division occurs, a bulge-like hyphal outgrowth is formed over the cross wall between the two daughter cells and provides a clamp connection to maintain the binucleate state of the resulting cells. These unique properties make genetic manipulation tools unsuitable or inefficient in mushrooms. This has further caused the slow development of biotechnological research of edible fungi in the past.

Articles in this Special Issue have demonstrated a favorable transition in the situation of adapting the genetic technology to mushrooms [1,2]. The CRISPR/Cas9 genome-editing system is a revolutionary technology and a powerful tool for precision molecular breeding. CRISPR/Cas9 systems were established in some edible fungi based on in vivo expressed Cas9 and guide RNA. Liu Xiaotian et al. employed plasmids harboring the codon-optimized Cas9 and dual sgRNAs to edit *pyrG* in *Flammulina filiformis*. It was the first successful CRISPR/Cas9 genome-editing system in *F. filiformis* [1]. Compared with this system, the in vitro assembled Cas9 and sgRNA ribonucleoprotein complexes (RNPs) have more advantages. Liu Jianyu et al. developed and optimized a CRISPR/Cas9 genome-editing method based on in vitro assembled RNP complexes in the same mushroom. The surfactant Triton X-100 played a critical role in this research [2]. These reports indicate that the development of edible mushroom biotechnology will accelerate along with this cutting-edge technology.

Most of the articles in this Special Issue are devoted to the bioactive components of *Ganoderma lingzhi* [3] and *G. lucidum* [4,5,6]. As traditional medicinal mushrooms with a history of more than 6800 years, the most concerned is the expression level and regulatory mechanism of genes related to the biosynthesis pathway of ganoderic acid [3,4,5,6]. Yan et al. investigated the metabolic flux distribution of three ganoderic acids. Intriguingly, the metabolic flux of ganoderic acids R was mostly benefited with a dramatical increase of 97.48% after adding oleic acid [6]. Mitochondria was proved that affected the content of polysaccharide and triterpenoid via starch and sucrose metabolism, steroid biosynthesis, and pentose and glucuronate interconversions [4]. The other two articles investigated the biosynthesis of these bioactive components through genome sequencing and transcriptome data [3,5].

Due to the commodity value of fruiting bodies, researchers frequently focus on morphological development and fungi–environment interactions. Guo et al. conducted the transcriptional and metabolic patterns of *Lentinula edodes* when the mycelial tissues were exposed to high temperature [7]. Yang et al. described a special link of spermidine and lysine which was probably involved in the development of mushroom fruiting body and in response to the multiple environmental factors [8]. Another study corroborated the long-chain fatty acid synthesis pathway which was responsible for stipe gradient elongation in *F. filiformis* [9]. Gong et al. offered a novel strategy of *Hypsizygus marmoreus* cultivation using vernalization-like low-temperature fruiting [10]. Li et al. speculated the horizontal transfer of functional 1-aminocyclopropane-1-carboxylate oxidase via analyzing phylogenesis [11]. Finally, a novel application of mushroom was conducted to enhance the nutritional value of highland barley via solid-state fermentation with *Agaricus sinodeliciosus* [12].

The Editors of this Special Issue express their sincere gratitude to all the authors who contributed to this Special Issue, as well as to MDPI’s staff for their professional help and efficient decisions.
